# Supplementary material for: Codon Bias Can Determine Sorting of a Potassium Channel Protein
Source: Cells. 2021 May 7;10(5):1128. doi: 10.3390/cells10051128 (PMC8151079; doi:10.3390/cells10051128)
Supplement: Supplementary file 1 [file cells-10-01128-s001.zip › cells-1151079-supplementary.pdf]

Supplement Figure 1:

Kcv_{wt}

ATGTTAGTGTTTAGTAAATTTCTAACGCGAACTGAACCATTCATGATACATCTCTTTATTCTCGCAATGTTCGTGATGA
TCTATAAATTCTTCCCGGGAGGGTTCGAAAATAACTTCTCTGTTGCAAACCCGGACAAAAAGGCATCATGGATAGATTG
TATATACTTCGGAGTAACGACACACTCTACTGTCGGATTCGGAGATATACTGCCAAAGACGACCGGCGCAAAGCTTTGT
ACGATAGCACATATAGTAACAGTGTTCTTCATCGTTCTAACTTTA

Kcv_{op}

ATGCTGGTGTTCTCTAAGTTCTTGACTAGAACTGAACCATTCATGATCCACTTGTTCATTTTGGCCATGTTCGTCATGA
TCTACAAGTTTTTTCCAGGTGGTTTCGAGAACAACTTCTCTGTTGCTAATCCAGATAAGAAGGCTTCTTGGATTGATTG
CATCTACTTCGGTGTTACTACTCATTCTACTGTTGGTTTCGGTGATATTTTGCCAAAAACTACTGGTGCTAAGTTGTGC
ACTATTGCTCATATCGTTACCGTTTTCTTCATCGTCTTGACCTTG

$Kesv_{wt}$

ATGTCCCGGCGACTGTTTGCGACTTGCGGCATCGCTATCGCGCTCAGGGGACTGGTGGTGAGCGGGGGCGTAAAAGAGA
TTGTATCGTTCAGGCCACTGATTGATACTTCGCTCGTCGGCGGAATATTGTCTAATCTGATTTTGCTCGTCGTTTTCGC
TGAACTTTATTGGCAGCTGGACCAAGGGGATGATCACACACACTTCGGCTTCTCGTCCGCGATCGACGCTTACTACTTC
AGTGCGGTCACGTCTTCCTCTGTCGGATACGGCGATTTGTTGCCGAAAACTCCGAAGGCAAAATTGCTTACCATCGCAC
ACATTTTGGCCATGTTCTTCGTGATGCTCCCCGTTGTCGCGAAGGCTCTCGAGAAG

$Kesv_{op}$

ATGAGCAGACGGCTGTTCGCCACCTGTGGAATCGCCATTGCCCTGCGGGGCCTGGTGGTGTCTGGCGGCGTGAAAGAAA
TCGTGTCCTTCCGGCCCCTGATCGACACCAGCCTCGTGGGAGGCATCCTGAGCAACCTGATCCTGCTGGTGGTGTTCGC
CGAGCTGTACTGGCAGCTGGACCAGGGCGACGACCACACCCACTTCGGCTTCAGCAGCGCCATCGACGCCTACTACTTC
AGCGCCGTGACCAGCAGCAGCGTGGGCTACGGCGACCTGCTGCCCAAGACCCCCAAGGCCAAGCTGCTGACAATCGCCC
ACATCCTGGCCATGTTCTTCGTGATGCTGCCCGTGGTGGCCAAGGCCCTGGAAAAG

>$Kesv_{ran}$

ATGTCGCGGCGGCTCTTTGCGACGTGCGGCATTGCTATAGCCTTAAGGGGCCTTGTCGTTTCCGGAGGCGTCAAGGAGA
TTGTCTCTTTTAGACCCCTCATTGACACCTCGTTGGTAGGTGGTATACTTTCGAATCTGATTCTGCTAGTCGTTTTTGC
AGAACTGTACTGGCAATTGGACCAGGGAGACGATCACACTCACTTCGGATTCAGTTCAGCCATAGATGCCTACTACTTC
TCAGCAGTGACGTCATCTAGTGTAGGTTACGGCGACTTGTTGCCCAAGACCCCGAAAGCAAAATTACTGACAATCGCGC
ACATCTTGGCTATGTTCTTCGTAATGCTTCCGGTTGTAGCGAAGGCTCTTGAAAAG

Linker 1

TTGTTGCCGAAAACTCCGAAGGCAAAATTGCTTACCATCGCACACATTTTGGCCATGTTCTTCGTGATGCTC
CCCGTTGTCGCGAAGGCTCTCGAGAAG

Linker 2

TTGTTGACTATTGCTCATATTTTGGCTATGTTTTTTGTTATGTTGCCAGTTGTTGCTAAGGCTTTGGAAAAG

eGFP

GGGATCCACCGGCCGGTCGCCACCATGGTGAGCAAGGGCGAGGAGCTGTTCACCGGGGTGGTGCCCATCCTG
GTCGAGCTGGACGGCGACGTAAACGGCCACAAGTTCAGCGTGTCCGGCGAGGGCGAGGGCGATGCCACCTAC
GGCAAGCTGACCCTGAAGTTCATCTGCACCACCGGCAAGCTGCCCGTGCCCTGGCCCACCCTCGTGACCACC
CTGACCTACGGCGTGCAGTGCTTCAGCCGCTACCCCGACCACATGAAGCAGCACGACTTCTTCAAGTCCGCC
ATGCCCGAAGGCTACGTCCAGGAGCGCACCATCTTCTTCAAGGACGACGGCAACTACAAGACCCGCGCCGAG
GTGAAGTTCGAGGGCGACACCCTGGTGAACCGCATCGAGCTGAAGGGCATCGACTTCAAGGAGGACGGCAAC
ATCCTGGGGCACAAGCTGGAGTACAACTACAACAGCCACAACGTCTATATCATGGCCGACAAGCAGAAGAAC
GGCATCAAGGTGAACTTCAAGATCCGCCACAACATCGAGGACGGCAGCGTGCAGCTCGCCGACCACTACCAG
CAGAACACCCCCATCGGCGACGGCCCCGTGCTGCTGCCCGACAACCACTACCTGAGCACCCAGTCCGCCCTG
AGCAAAGACCCCAACGAGAAGCGCGATCACATGGTCCTGCTGGAGTTCGTGACCGCCGCCGGGATCACTCTC
GGCATGGACGAGCTGTACAAGTAA

Figure S1: Components of channel-linker-GFP constructs

Gene sequences of wt (wt), codon optimized (op) and a random mix of rare and non-rare codons (ran) for Kcv and Kesv channels as well as gene sequence of linkers (1 and 2) and eGFP.

Supplement Figure 2:

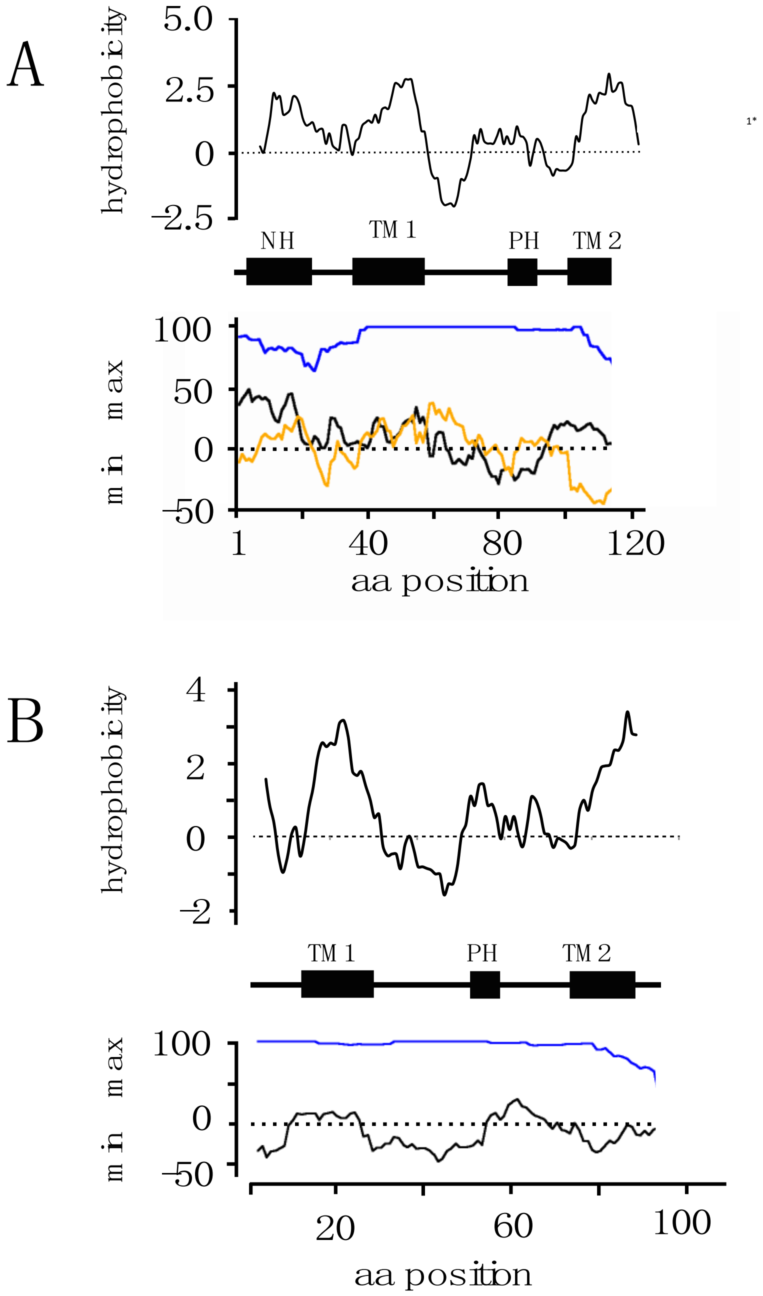

Fig. S2: Schematic domain architectures and codon usage plots of Kesv and Kcv.
Kyte-Doolittle plots of Kesv **(A)** and Kcv **(B)** channels (top) and predicted location of -helices including the N-terminal helix (NH), outer (TM1) and inner (TM2) transmembrane domain and pore helix (PH) (central panel). Lower panel shows calculation of mean distribution of most common (max) and least common codons (min) in human cells for wt gene (Kesv$_{wt}$ and Kcv$_{wt}$ black) codon optimized gene (Kesv$_{op}$, Kcv$_{op}$: blue) and randomized gene (Kesv$_{ran}$: orange) calculated with %MinMax algorithm (http://www.codons. org/Info.html). The plot provides information on whether the entire gene sequence for channel plus linker and GFP is composed of most common codons possible (=100%) or least common codons possible (−100%).

Supplement Figure 3:

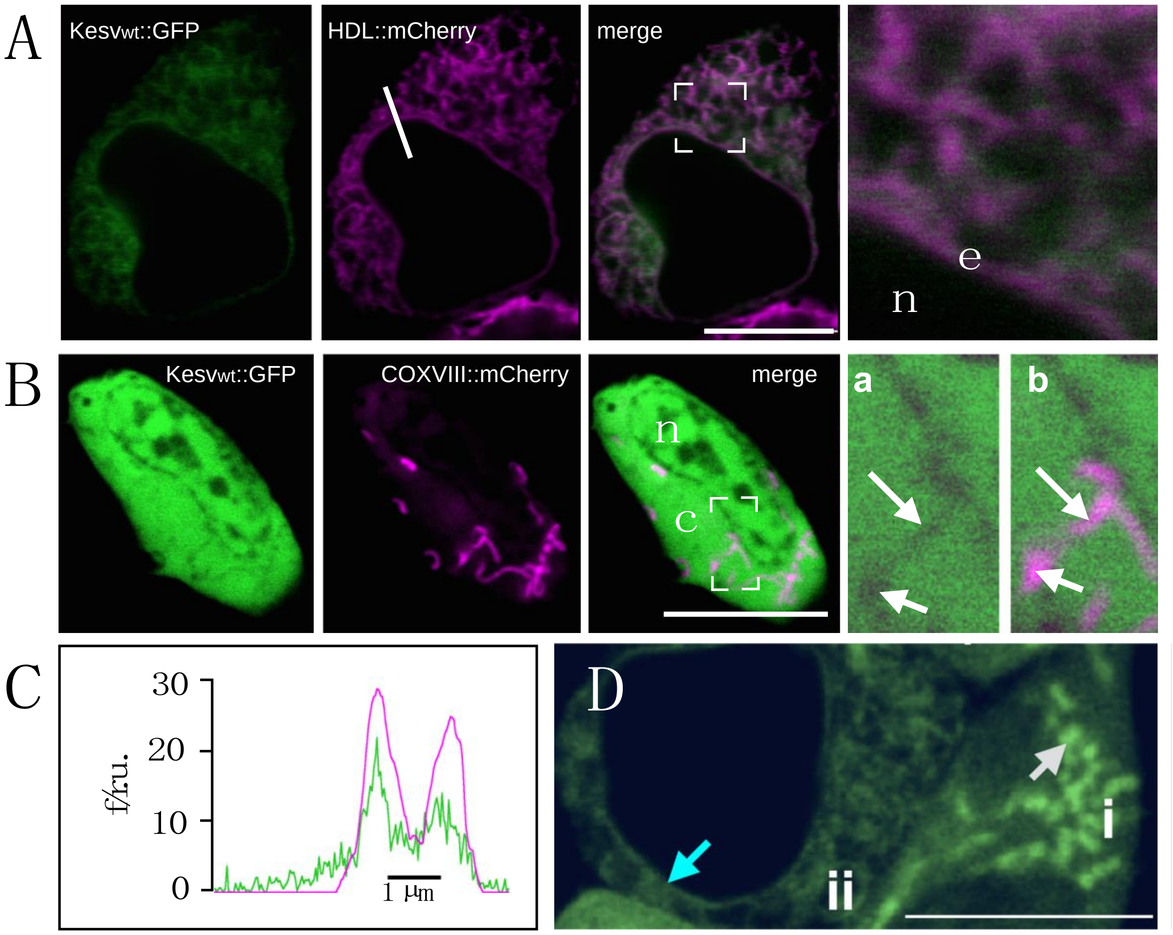

Figure 3S. Sorting pattern of Kesv channel.

A,B Fluorescent images of HEK293 cells transfected with Kesv$_{wt}$. Images show: fluorescence of GFP tagged Kesv channel (green, first column), fluorescence from mitochondrial marker COXVII::mCherry or ER marker HDEL::mCherry (magents, second column) and overlay of magenta and green channels (third column). A magnification from areas marked in overlay images is shown in the fourth column. Letters in the images refer to cytosol (c), nucleus (n), mitochondria (m), and ER (e). Magnified images in 2nd row show the same section from the GFP channel only (a) and from overlay of green and magenta channel (b). Arrows indicate areas which are spared from green fluorescence (a) and which contain a signal from the magenta signal in the overlay (b). **C** reports the co-localization of green and magenta channels at position along the line shown in magenta channel in **A**. **D**. Fluorescence image of two adjacent HEK293 cells transfected with Kesv$_{wt}$ gene. In one cell (i) the protein is sorted to the mitochondria indicated by the white arrow. In the second cell (ii) the channel appears in the SP, which is characterized by typical net-like structure and the peri-nuclear ring (blue arrow). Scale bars: 10 µm.

Supplement Figure 4:

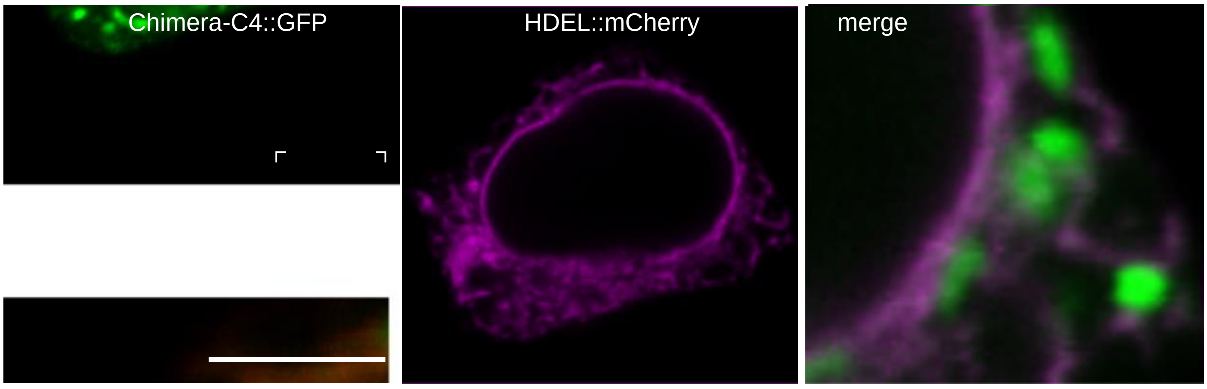

Figure S4: Dual sorting of Chimera C4 in the same HEK293 cell into SP and mitochondria. Fluorescent image of HEK293 cell with fluorescence from GFP-tagged channel (green, left panel) and fluorescence of ER marker HDEL::mCherry (magenta, central image). Magnification of overlay of green and magenta channels with colocalization between ER and GFP fluorescence as well as bright GFP fluorescence in mitochondria. Scale bar 10 μm.

Supplement Figure 5:

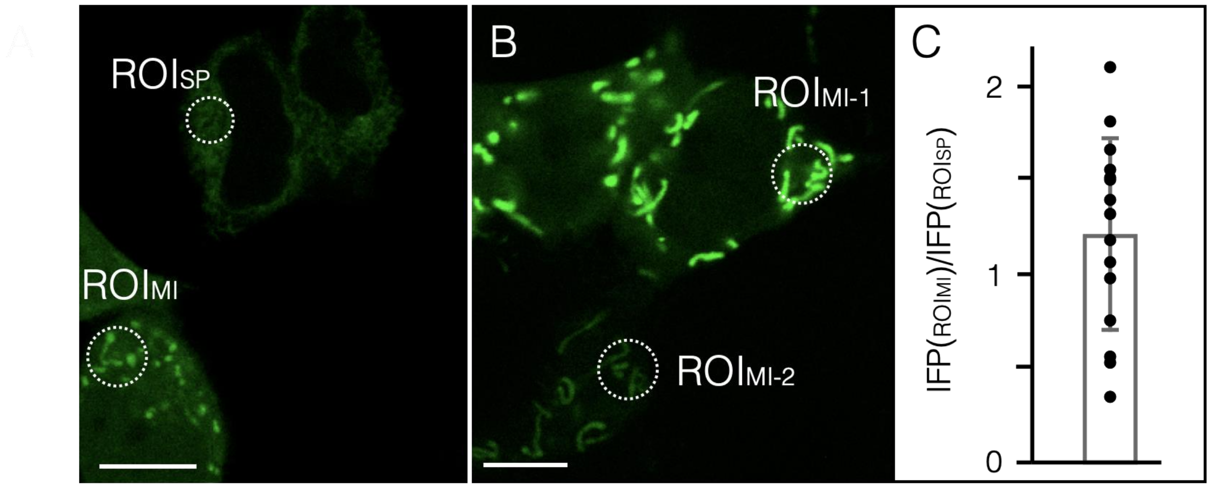

Figure S5: Comparative GFP fluorescence intensity from Kesv in adjacent HEK293 with either mitochondria or SP sorting. A Fluorescent image of HEK293 cells with fluorescence from GFP-tagged Kesv channel. In one cell GFP is present in mitochondria (down) in two other cells in SP (up). Dashed circles indicate regions of interest (ROI) covering mitochondria (ROI$_{MI}$) or SP (ROI$_{SP}$). **B** Same as in (A) but with cells showing Kesv in mitochondria with high (MI-1) or low (MI-2) fluorescence. **C** Ratios of integrated fluorescence density per ROI area from ROI$_{SP}$ and ROI$_{MI}$ (IFD(ROI$_{MI}$)/IFD(ROI$_{SP}$)). Mean value ± SD and individual data from 15 cell pairs as in A (circles). Scale bars: 10 μm.
